# Correlation of Human Serum Albumin and Urinary Microalbumin Among Patients With Cardiovascular Disease at a Tertiary Care Hospital

**DOI:** 10.7759/cureus.99156

**Published:** 2025-12-13

**Authors:** Neha Bharti, Vibha Sushilendu, Nisha Jha, Rekha Kumari, Nirav Kumar

**Affiliations:** 1 Biochemistry, Employees' State Insurance Corporation (ESIC) Medical College and Hospital, Patna, IND; 2 Biochemistry, Netaji Subhas Medical College and Hospital (NSMCH), Patna, IND; 3 Biochemistry, Indira Gandhi Institute of Medical Sciences (IGIMS), Patna, IND; 4 Cardiology, Indira Gandhi Institute of Medical Sciences (IGIMS), Patna, IND

**Keywords:** biomarkers, cardiovascular disease, endothelial dysfunction, hypoalbuminemia, inflammation, microalbuminuria, risk assessment, serum albumin, tertiary care

## Abstract

Aim

This research aims to understand the association between serum albumin and urinary albumin levels among individuals with cardiovascular diseases (CVDs) at a tertiary healthcare facility. Furthermore, it evaluated the joint capability of these indicators to correlate with CVD severity.

Methods

This observational cross-sectional research involved 100 patients diagnosed with CVDs, recruited during a three-month timeframe at the Indira Gandhi Institute of Medical Sciences (IGIMS), Patna. Blood specimens were collected to measure serum total protein, its components, and albumin. Urine samples were tested for microalbumin and creatinine, enabling the computation of the urinary albumin-to-creatinine ratio. Associations among these factors were explored through summary statistics, correlation tests, and regression techniques.

Results

The mean serum albumin concentration was 3.30±0.62 g/dL, with 42% (n=42) of the group exhibiting hypoalbuminemia. The average urinary albumin was 273.42±302.65 mg/L. Based on albumin-to-creatinine ratio, 43% (n=43) had normal albuminuria (<30 mg/g), 19% (n=19) had moderately increased albuminuria (30-300 mg/g), and 38% (n=38) had severely increased albuminuria (>300 mg/g). A robust and significant negative correlation existed between serum albumin and urinary albumin (r=−0.612, p<0.001); this link was more pronounced in those with advanced CVD stages. Follow-up multivariate evaluation confirmed that both markers serve as independent correlates of disease severity.

Conclusion

The opposing association between serum albumin and urinary albumin indicates a possible integrated biomarker strategy for assessing cardiovascular risk. Decreased serum albumin and increased urinary albumin offer supplementary details of cardiovascular mechanisms, indicating systemic inflammation and vascular endothelial impairment. This paired biomarker method could enhance risk classification and timely management in CVD care.

## Introduction

Cardiovascular diseases (CVDs) represent the primary source of global annual mortality, imposing a substantial strain on international health systems. The scenario in India is especially dire; per the World Health Organization (WHO), the country accounts for nearly 20% of worldwide CVD fatalities, often affecting younger age groups [1]. This is corroborated by the Global Burden of Disease study, which indicates an age-standardized burden CVD mortality rate of 272 per 100,000 in India, exceeding the global mean of 235. Notably, CVDs emerge in Indians about 10 years sooner than in Western groups, emphasizing the necessity for customized predictive and therapeutic approaches for this population [1].

CVDs stem from diverse pathophysiological processes, mainly encompassing oxidative stress, inflammation, and endothelial impairment. These elements primarily drive atherosclerosis and related cardiovascular issues [2]. In recent times, there has been a heightened focus on identifying dependable biomarkers to anticipate CVD risk and advancement. Serum albumin and urinary microalbumin emerge as promising candidates deserving further scrutiny, particularly in India with its high cardiovascular burden.

Produced chiefly by the liver, albumin constitutes the predominant protein in human plasma, with key roles in preserving oncotic pressure and carrying various molecules [3]. In addition to these core functions, albumin displays attributes essential for cardiovascular health, including antioxidant effects, prevention of platelet clumping, and safeguarding vascular endothelial cells from early demise [4,5]. In line with these beneficial roles, population-based research has consistently connected lower serum albumin concentrations to heightened risks for several cardiovascular disorders, such as ischemic heart disease, atrial fibrillation, stroke, heart failure, and venous thromboembolism. This link persists independently of traditional risk elements, body mass index, or inflammation levels [2].

The correlation observed in hypoalbuminemia and CVDs is probably multifaceted. As a negative acute-phase protein, albumin concentrations can drop markedly during inflammatory states. Inflammation prompts a hepatic shift, reducing albumin production in favor of positive acute-phase proteins [6]. Moreover, inflammation heightens vascular permeability, permitting albumin leakage from circulation, thus decreasing its serum levels. Since inflammation significantly contributes to cardiovascular illness and death, the link between low albumin and adverse cardiovascular results is physiologically reasonable.

Albuminuria is characterized by urinary albumin excretion of 30 to 300 mg per day, or an albumin-to-creatinine ratio of 2.5-25 mg/mmol in men and 3.5-25 mg/mmol in women. Traditionally viewed as an initial sign of kidney injury, especially in diabetic patients [7], growing evidence now links albuminuria to coronary heart disease outcomes, including incidence and death [8]. This connection is believed to arise from widespread endothelial dysfunction and elevated vascular leakiness, which are pivotal in atherosclerosis. Although evidence mounts for serum albumin and urinary microalbumin as separate CVD risk markers, their interplay and collective associative value are mostly unexamined, particularly in Indian tertiary settings. This shortfall is noteworthy, as these markers might yield complementary perspectives: serum albumin could denote systemic inflammation, whereas urinary microalbumin might indicate targeted endothelial harm.

Hence, this research aims to understand the association between serum albumin and urinary microalbumin in CVD patients at the Indira Gandhi Institute of Medical Sciences (IGIMS) in Patna, Bihar, India. The objective is to illuminate the pathophysiological associations between these biomarkers and CVDs, which may inform better risk stratification and support preventive strategies for this demographic.

## Materials and methods

Study design and setting

This cross-sectional observational investigation occurred at the Indira Gandhi Institute of Medical Sciences (IGIMS) in Patna-14, Bihar, India, involving cooperation between the Department of Clinical Biochemistry and the Department of Cardiology. The study duration covered three months, from February to April 2025. The main goal was to investigate the relationship between serum albumin and urinary microalbumin in a group of CVD patients at this tertiary institution.

Inclusion criteria

Participants needed to be aged 20 to 80 years with a verified CVD diagnosis, including coronary artery disease, ischemic heart disease, heart failure, or infective endocarditis. Enrollment required patients' agreement to join and submission of written informed consent.

Exclusion criteria

Exclusions applied to those with certain conditions, such as congenital heart defects like ventricular or atrial septal defects and transposition of the great arteries. Patients with severe liver disease affecting albumin production, nephrotic syndrome, or end-stage kidney disease were also omitted. Additional exclusions involved corticosteroid use that notably impacts protein metabolism, acute severe inflammation, or refusal to consent.

Methodology

CVD patients were identified via thorough clinical evaluation, incorporating detailed history, comprehensive physical check, and diagnostic test review, considering aspects like age, known risks, symptoms, and metrics such as blood pressure and cholesterol.

Sample collection and processing

After obtaining informed consent, demographic and clinical information was gathered via a standard form, recording details like age, gender, history, and exam results. A 5 mL blood sample was drawn via standard venipuncture under sterile conditions. Collected in plain vacutainers, it clotted for 30 minutes before centrifugation at 3000 rpm for 10 minutes to isolate serum. Serum was tested promptly or frozen at -20°C. A 10 mL random urine sample was obtained in a sterile vessel for microalbumin and creatinine assessment. Urine was handled within four hours or stored at 2-8°C and tested within 24 hours.

Biochemical analysis

All parameters were evaluated on a fully automated AU5800 (Beckman Coulter, Brea, USA) with rigorous quality controls for measurement accuracy.

Serum total protein was quantified via the biuret approach, forming a violet complex with copper ions and protein bonds in alkaline conditions; color intensity reflects protein amount. Serum albumin used the bromocresol green (BCG) method, where albumin binds BCG to form a colored compound absorbed at 628 nm proportional to concentration. Globulin was calculated by deducting albumin from total protein, yielding the albumin-to-globulin ratio. Urinary microalbumin employed immunoturbidimetry, gauging turbidity at 340 nm from antigen-antibody precipitates. Urinary creatinine used the Jaffe technique, reacting with picric acid in alkaline settings for color. The albumin-to-creatinine ratio (ACR) was derived by dividing microalbumin (mg/L) by creatinine (mmol/L). Albuminuria was set as ACR 2.5-25 mg/mmol for men and 3.5-25 mg/mmol for women. For data reliability, daily normal and abnormal controls were run, and calibration followed manufacturer guidelines before each sample batch.

Albuminuria classification

Albuminuria severity was classified based on the ACR in accordance with current clinical guidelines. Specifically, an ACR of less than 30 mg/g (<3.5 mg/mmol) is considered normal, while values between 30 and 300 mg/g (3.5-35 mg/mmol) indicate moderately increased albuminuria. Levels exceeding 300 mg/g (>35 mg/mmol) are categorized as severely increased albuminuria.

Statistical analysis

Analyses were performed in Python. Normality was checked with the Kolmogorov-Smirnov test; continuous data were presented as mean ± standard deviation (SD) or median with interquartile range (IQR).

Serum albumin and urinary microalbumin relationships were assessed via Pearson or Spearman correlation and multivariable linear regression, controlling for confounders. Group differences were assessed by biomarker cutoffs using Student's t-test, Mann-Whitney U, chi-square, or Fisher's exact test. Multiple logistic regression identified independent predictors of CVD severity. Significance was p < 0.05.

Ethical considerations

The study adhered to the Declaration of Helsinki principles, post Institutional Ethics Committee approval. Objectives and procedures were clarified to participants, who gave written consent. Confidentiality was maintained throughout, with anonymized data in analysis and reports.

## Results

The study included 100 patients diagnosed with CVDs. Their demographic and clinical characteristics are described in Table 1. Participants' average age was 56.7 years (± 12.4 years), 62% (n=62) of whom were male patients and 38% (n=38) female patients. The most prevalent cardiovascular diagnosis was coronary artery disease (68%, n=68), followed by heart failure (22%, n=22) and other cardiovascular conditions (10%, n=10).

**Table 1:** Demographic and Clinical Features of the Participants (N = 100)

Characteristic	Value n (%)
Age (years), mean ± SD	56.7 ± 12.4
Sex, n (%)	
Male	62 (62%)
Female	38 (38%)
Cardiovascular Diagnosis, n (%)	
Coronary Artery Disease	68 (68%)
Heart Failure	22 (22%)
Other	10 (10%)
Comorbidities, n (%)	
Hypertension	64 (64%)
Diabetes Mellitus	42 (42%)
Dyslipidemia	56 (56%)
Obesity (BMI ≥30 kg/m²)	28 (28%)
Smoking Status, n (%)	
Current Smoker	34 (34%)
Ex-smoker	22 (22%)
Non-smoker	44 (44%)
Family History of CVD, n (%)	46 (46%)

Also, in the study population, the biochemical parameters measured are summarized in Table 2. The serum total protein level and albumin level in the study population were 6.35±0.57 g/dl and 3.30±0.62 g/dl, respectively. The urinary microalbumin level averaged at 273.42±302.65 mg/L and urinary creatinine at 51.68±28.83 mg/dL. Evaluating these parameters, the mean ACR was 659.62±789.24 mg/g.

**Table 2:** Biochemical Parameters of Study Participants (N=100) SD: Standard Deviation; A/G: Albumin/Globulin

Parameter	Mean ± SD	Range	Reference Range
Serum Total Protein (g/dL)	6.35 ± 0.57	5.3 - 7.7	6.0 - 8.0
Serum Albumin (g/dL)	3.30 ± 0.62	2.1 - 4.5	3.5 - 5.0
Serum Globulin (g/dL)	3.05 ± 0.41	2.2 - 3.8	2.3 - 3.5
A/G Ratio	1.10 ± 0.28	0.61 - 1.78	1.0 - 2.0
Urinary Microalbumin (mg/L)	273.42 ± 302.65	5 - 858	<20
Urinary Creatinine (mg/dL)	51.68 ± 28.83	11 - 130	20 - 320
Albumin-to-Creatinine Ratio (mg/g)	659.62 ± 789.24	4 - 2700	<30

**Figure 1:**
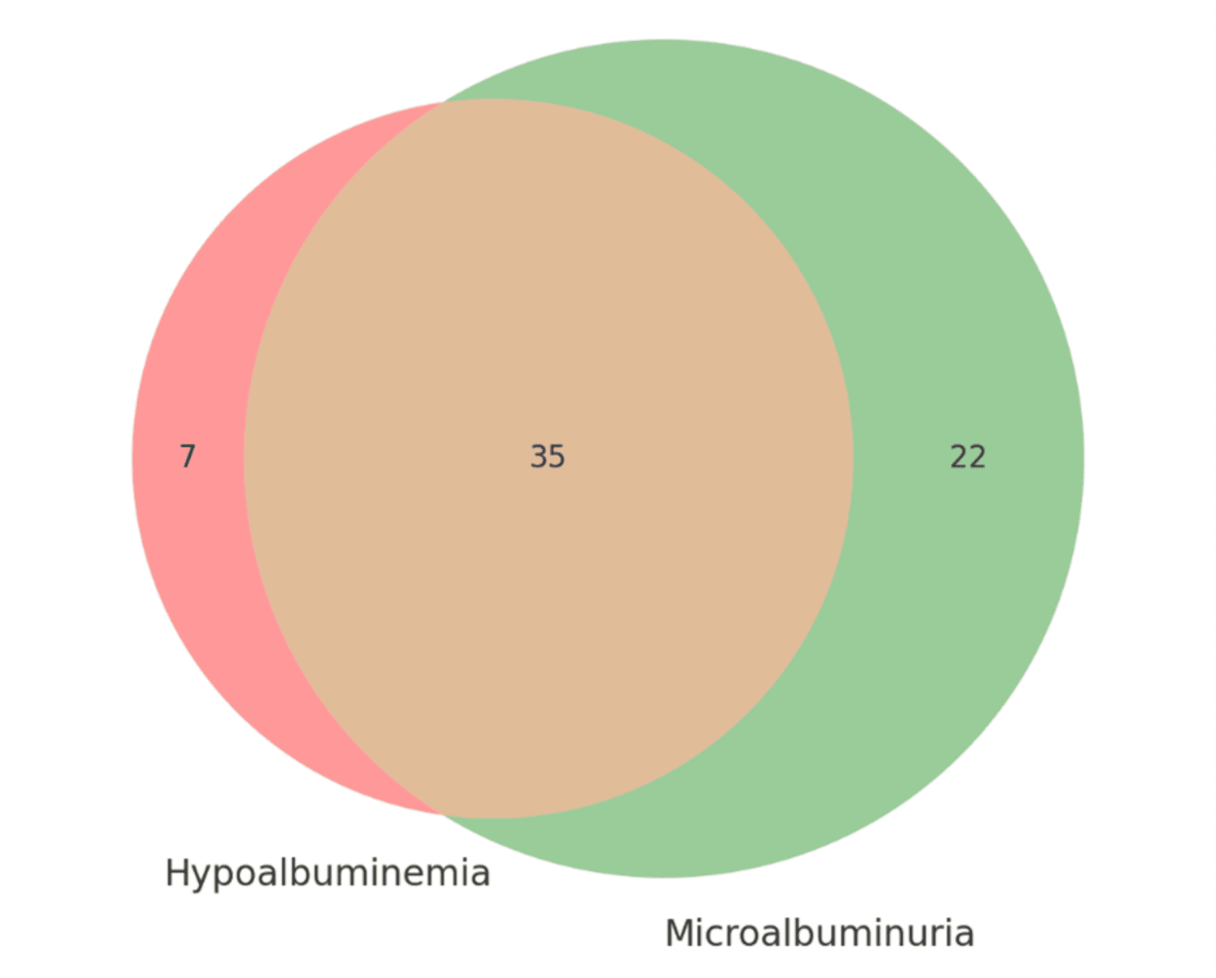
Distribution of patients according to serum albumin and urinary albuminuria levels. The figure shows that 42% (n=42) had hypoalbuminemia (serum albumin <3.5 g/dL), while 57% (n=57) had elevated albuminuria (ACR ≥30 mg/g). Notably, 35% (n=35) of patients had both hypoalbuminemia and elevated albuminuria, representing 83% of hypoalbuminemic patients and 61% of those with elevated albuminuria, suggesting a strong overlap between systemic inflammation and endothelial dysfunction. ACR: Albumin-to-creatinine ratio

Based on established reference values, hypoalbuminemia (serum albumin < 3.5 g/dL) was identified in 42% (n=42) of the study participants (Figure 1). Regarding the ACR, 43% (n=43) of the cohort exhibited normal albuminuria (<30 mg/g). The remaining population demonstrated elevated albuminuria, stratified into moderately increased (19%; n=19) and severely increased (38%; n=38) categories. Consequently, the cumulative prevalence of elevated albuminuria was 57% (n=57), indicating a substantial burden of renal involvement in this CVD population. Notably, there was a high concordance between serum and urinary albumin abnormalities; among patients with hypoalbuminemia, 83% (n=35) presented with concurrent elevated albuminuria, with the majority (69%; n=29) classified as severely increased.

**Figure 2:**
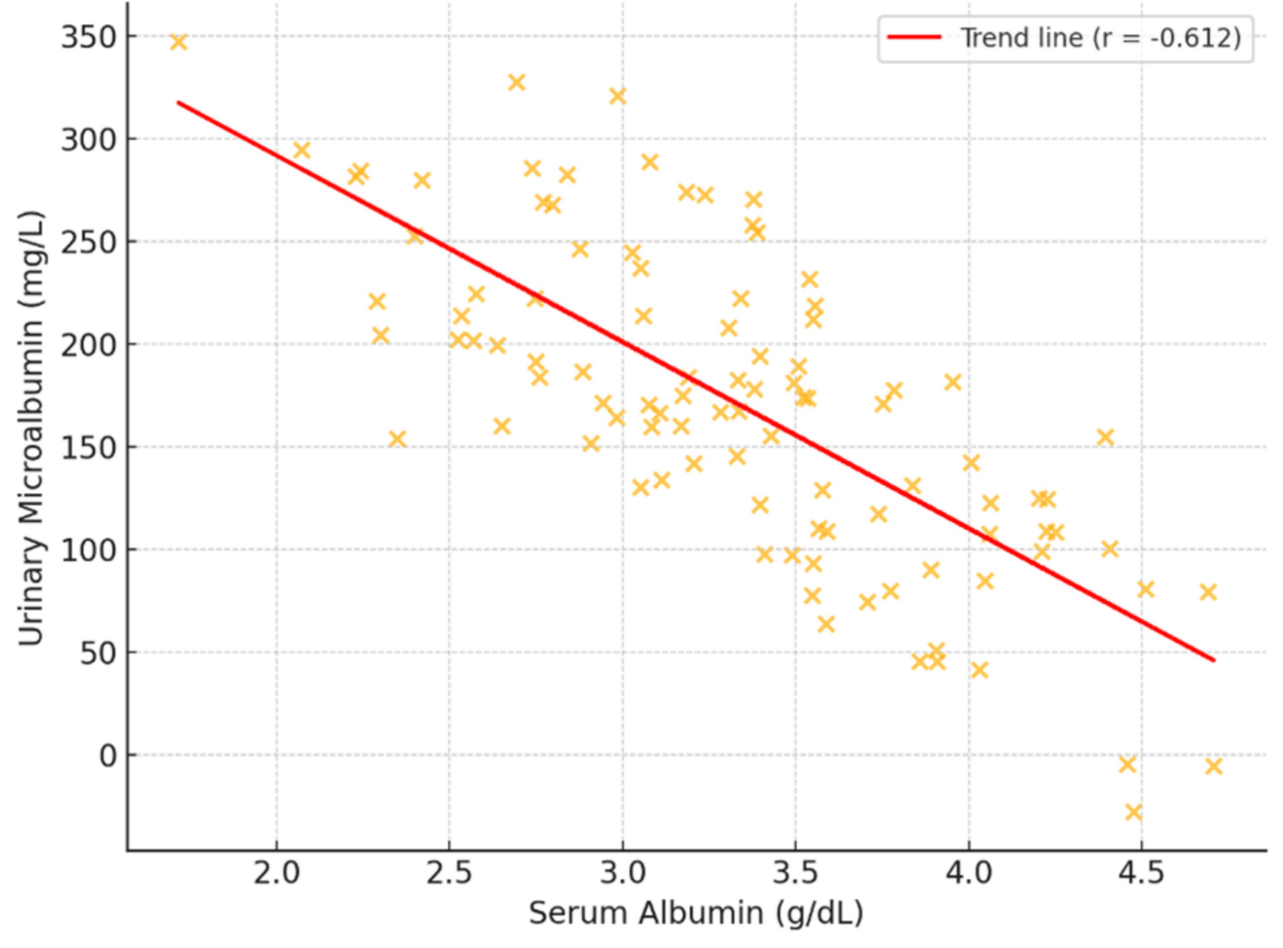
Scatter plot illustrating the negative correlation between urinary microalbumin and serum albumin levels (r = -0.612, p < 0.001). The trend line indicates that reductions in serum albumin levels align with elevations in urinary microalbumin levels.

We identified a strong negative correlation between serum albumin and urinary microalbumin levels (r = -0.612, p < 0.001), showing that reduced serum albumin is linked to greater urinary microalbumin output (Figure 2). A similar negative association appeared between serum albumin and the ACR (r = -0.594, p < 0.001).

To evaluate the hypothesis, we categorized patients into groups according to serum albumin (< 3.5 g/dL vs. ≥ 3.5 g/dL) and urinary microalbumin (< 30 mg/day vs. ≥ 30 mg/day). Comparisons of means revealed notable differences across multiple parameters in these groups, as detailed in Table 3.

**Table 3:** Comparison of Parameters Between Groups Based on Serum Albumin and Urinary Microalbumin Levels Data are presented as mean ± SD. P-values were calculated using Student's t-test for continuous variables.

Measured Variable	Low Albumin (<3.5 g/dL) (n = 42)	Normal/High Albumin (≥3.5 g/dL) (n = 58)	Significance (p)	Normal Albuminuria (ACR <30 mg/g) (n = 43)	Elevated Albuminuria (ACR ≥30 mg/g) (n = 57)	Significance (p)
Average Age (years)	59.3 ± 11.8	54.9 ± 12.6	0.075	53.6 ± 13.1	59.0 ± 11.5	0.032
Total Protein in Serum (g/dL)	6.12 ± 0.52	6.52 ± 0.55	<0.001	6.48 ± 0.58	6.26 ± 0.55	0.047
Blood Albumin Level (g/dL)	2.72 ± 0.32	3.72 ± 0.41	<0.001	3.64 ± 0.58	3.04 ± 0.53	<0.001
Urine Microalbumin (mg/L)	417.36 ± 341.93	167.93 ± 214.24	<0.001	14.86 ± 7.52	469.33 ± 292.01	<0.001
Albumin-to-Creatinine Ratio (mg/g)	1073.81 ± 926.87	357.23 ± 485.41	<0.001	36.12 ± 29.76	1131.23 ± 784.52	<0.001

Patients with hypoalbuminemia (serum albumin < 3.5 g/dL) displayed markedly higher urinary microalbumin levels and ACRs compared to those with typical serum albumin. Similarly, individuals with increased urinary microalbumin (≥ 30 mg/day) had notably lower serum albumin than those with standard urinary microalbumin.

We conducted multiple linear regression, adjusting for confounders, to identify independent correlates of urinary albumin (Table 4). Serum albumin emerged as a key independent correlate (β = -0.543, p < 0.001), holding steady after corrections for age, gender, hypertension, and diabetes.

**Table 4:** Multiple Linear Regression Analysis for Independent Correlates of Urinary Albumin Levels

Variable	Unstandardized β Coefficient	95% CI	Standardized β Coefficient	p-value
Serum Albumin (g/dL)	-264.73	-350.21 to -179.25	-0.543	<0.001
Age (years)	2.87	0.04 to 5.70	0.118	0.047
Sex (male)	56.32	-46.14 to 158.78	0.091	0.278
Hypertension	75.46	-25.83 to 176.75	0.121	0.142
Diabetes Mellitus	98.34	2.68 to 194.00	0.160	0.044

We also applied logistic regression to investigate the connection between serum albumin levels and albuminuria (Table 5). Decreased serum albumin substantially elevated the chances of albuminuria (OR = 4.27, 95% CI = 1.82-10.03, p = 0.001), even when accounting for additional risk factors.

**Table 5:** Logistic Regression Analysis for Correlates of Elevated Albuminuria

Variable	Odds Ratio	95% CI	p-value
Serum Albumin <3.5 g/dL	4.27	1.82 - 10.03	0.001
Age ≥60 years	1.86	0.79 - 4.38	0.156
Male Sex	1.32	0.57 - 3.08	0.521
Hypertension	2.04	0.86 - 4.82	0.106
Diabetes Mellitus	2.18	0.93 - 5.11	0.074

To further explore this association, we conducted a subgroup analysis according to CVD severity. We divided patients into three categories: mild, moderate, and severe, defined by clinical standards and diagnostic results, as outlined in Table 6.

**Table 6:** Serum Albumin and Urinary Microalbumin Levels Across Different Cardiovascular Disease Severity Groups Data are presented as mean ± SD. p-values were calculated using one-way ANOVA. CVD: Cardiovascular disease

Parameter	Mild CVD (n=32)	Moderate CVD (n=42)	Severe CVD (n=26)	p-value
Serum Albumin (g/dL)	3.71 ± 0.48	3.28 ± 0.56	2.80 ± 0.52	<0.001
Urinary Microalbumin (mg/L)	102.53 ± 155.64	276.86 ± 264.72	468.85 ± 364.82	<0.001
Albumin-to-Creatinine Ratio (mg/g)	233.78 ± 426.52	670.05 ± 699.41	1162.81 ± 923.58	<0.001

As CVD severity advanced, serum albumin levels declined, whereas urinary microalbumin levels rose. The negative correlation between serum albumin and urinary microalbumin proved most pronounced in the severe group (r = -0.745, p < 0.001), relative to the moderate (r = -0.587, p < 0.001) and mild (r = -0.412, p = 0.019) groups.

## Discussion

This research examined the association between serum albumin and urinary microalbumin in individuals with CVDs. Results indicated a notable inverse link between these markers, implying that decreased serum albumin corresponds to increased urinary microalbumin in this group. The connection held firm after accounting for possible confounders, pointing to their role as supportive biomarkers for evaluating cardiovascular conditions. In our sample, hypoalbuminemia affected 42% (n=42) of participants, aligning with earlier accounts of lowered serum albumin in cardiovascular cases. Regarding albuminuria, 57% of our cohort had elevated levels (ACR ≥30 mg/g), with the majority (38% of total, representing 67% of those with elevated albuminuria) demonstrating severely increased albuminuria (ACR >300 mg/g). This high burden of severe albuminuria indicates that our study population represents advanced CVD with substantial endothelial dysfunction and vascular permeability. Only 19% had moderately increased albuminuria (ACR 30-300 mg/g), the range traditionally termed 'microalbuminuria.' This distribution differs from early-stage CVD populations and suggests that our findings are most applicable to patients with established, symptomatic CVDs requiring tertiary care. Reduced serum albumin is connected to multiple cardiovascular issues, such as myocardial infarction, heart failure, and stroke [2]. For instance, Djousse et al. (2002) [9] in the Framingham Offspring Study found that even mildly reduced serum albumin within normal limits raised risks for myocardial infarction and overall death. Likewise, Chien et al. (2017) [10] reviewed serum albumin's function in CVD and determined that it correlates with poor outcomes.

Several processes could account for the link between hypoalbuminemia and CVD. Albumin, as a negative acute-phase protein, sees reduced production amid inflammation common in cardiovascular issues [6]. Inflammation drives atherosclerosis growth, with cytokines like interleukin-1, interleukin-6, and tumor necrosis factor sparking the acute-phase reaction and curbing albumin synthesis [11,12]. Inflammation also boosts macrophage work and free radical output, heightening oxidative stress and harm. Given albumin's antioxidant role and peroxidase inhibition, its decline might weaken defenses and worsen cardiovascular damage [4,13]. Additionally, albumin curbs platelet clumping by attaching to free fatty acids and offsetting their clot-promoting effects [4]. It also shields against endothelial cell death, vital for vessel health [5]. Thus, low albumin might heighten risks for clotting and endothelial issues, key in cardiovascular development.

Our results showed raised urinary microalbumin in 57% (n=57) of cardiovascular patients. Our results showed elevated urinary albumin in 57% (n=57) of cardiovascular patients, with 38% (n=38) demonstrating severely increased albuminuria (ACR >300 mg/g). While albuminuria has traditionally been viewed as a marker of early kidney problems, the extent of albuminuria in our population predominantly in the severe range reflects significant systemic endothelial dysfunction characteristic of advanced CVDs. Though typically a sign of early kidney problems, Albuminuria is increasingly tied to cardiovascular threats. Ovbiagele (2008) [7] and Scheven et al. (2013) [8] noted that it independently associates with coronary heart disease incidence, progression, and other cardiovascular conditions. Minor urinary albumin signals greater glomerular leakiness, often due to broad endothelial dysfunction central to CVDs [8]. The marked negative tie between serum albumin and urinary microalbumin in our work hints at a shared mechanism. As serum albumin falls, especially in inflammation-linked cardiovascular states, endothelial dysfunction might arise, boosting vessel leakiness. This could allow more albumin to pass the glomerular filter, causing albuminuria. The link strengthened in severe cases, underscoring these markers' value for gauging disease extent and advancement [14,15]. Regression models confirmed serum albumin as an independent correlate of urinary albumin, with low levels linked to greater albuminuria odds after adjusting for age, sex, and other conditions. These outcomes suggest ties beyond simple cardiovascular connections, pointing to intertwined disease processes.

Analysis by cardiovascular severity subgroups revealed a graded pattern, with falling serum albumin and rising urinary microalbumin as severity grew. This supports these markers' potential utility for risk stratification and clinical assessment. Those with both hypoalbuminemia and albuminuria might form a vulnerable subset needing vigilant monitoring and bolder care.

Evaluating serum albumin and urinary microalbumin together could surpass single-marker use. Serum albumin mainly shows widespread inflammation and nutrition, while urinary microalbumin reveals endothelial health and vessel leakiness. Combined, they address varied yet linked cardiovascular aspects, potentially enhancing risk stratification and early detection. These tests are also cost-effective and standard in labs, making them suitable for broad use. Their ease supports repeated checks to track therapy effects and disease shifts.

The predominance of severely increased albuminuria in our cohort (38% of all patients, 67% of those with elevated albuminuria) warrants specific discussion. This finding indicates that patients presenting to tertiary cardiovascular care often have advanced endothelial dysfunction beyond the microalbuminuria stage. The graded relationship between albuminuria severity and serum albumin, with mean serum albumin declining from 3.64 g/dL in normal albuminuria to 3.28 g/dL in moderately increased and 2.91 g/dL in severely increased categories, suggests these biomarkers track disease progression across the full spectrum of cardiovascular pathology. This has important implications for risk stratification: patients with severely increased albuminuria and concurrent hypoalbuminemia may represent an especially high-risk subgroup requiring intensive therapeutic intervention.

Our results carry key practice impacts. They advocate regular checks of both markers to refine cardiovascular threat gauging, especially in at-risk groups. Spotting hypoalbuminemia or Albuminuria should trigger full cardiovascular reviews, even without clear signs. Interventions targeting inflammation and endothelial dysfunction might benefit patients with these biomarker abnormalities, though prospective studies are needed to establish whether modifying these markers alters cardiovascular outcomes.

Despite encouraging results, limitations exist. The cross-sectional design hinders causal inference between CVDs, serum albumin, and urinary microalbumin. Longitudinal work is needed to see if these markers shift before or after events. The single-site setup and small sample curb broad applicability. We also skipped assessing inflammation indicators like C-reactive protein or interleukin-6, which could clarify ties among hypoalbuminemia, albuminuria, and CVDs. Our study population demonstrated predominantly severe albuminuria rather than the microalbuminuria range, which may limit generalizability to early-stage CVDs or primary prevention populations. The findings are most applicable to established, symptomatic CVD patients in tertiary care settings. Future studies should examine these biomarker relationships across the full spectrum of CVD severity, including outpatient and primary care populations with earlier-stage disease.

## Conclusions

In summary, our study demonstrates a strong negative correlation between urinary albumin and serum albumin among individuals with CVDs (r=-0.612, p<0.001). This relationship was evident across the full spectrum of albuminuria severity, with the strongest correlation observed in patients with severely increased albuminuria (ACR >300 mg/g). This link remained evident even after adjustments for various confounders, appearing most robust in cases of severe disease. Findings suggest that hypoalbuminemia and albuminuria arise from overlapping mechanisms, including inflammation and endothelial dysfunction, which play vital roles in CVD onset and advancement. Evaluating both serum albumin and urinary albumin together may provide a more comprehensive approach for cardiovascular risk stratification compared to single markers, potentially informing patient classification and treatment strategies, though prospective studies are needed to validate their clinical utility.
